# Older patients’ experiences with a shared decision-making process on choosing dialysis or conservative care for advanced chronic kidney disease: a survey study

**DOI:** 10.1186/s12882-019-1423-x

**Published:** 2019-07-16

**Authors:** Wouter R. Verberne, Wanda S. Konijn, Karen Prantl, Janneke Dijkers, Margriet T. Roskam, Johannes J. M. van Delden, Willem Jan W. Bos

**Affiliations:** 1 Department of Internal Medicine, St Antonius Hospital, Koekoekslaan 1, 3435 CM Nieuwegein, The Netherlands; 2 Dutch Kidney Patients Association (NVN), Bussum, The Netherlands; 3 University Medical Centre Utrecht, Julius Centre for Health Sciences and Primary Care, Utrecht, The Netherlands; 4 Department of Internal Medicine, Leiden University Medical Center, Leiden, The Netherlands

**Keywords:** Shared decision making, Chronic kidney failure, Renal dialysis, Conservative treatment, Aged

## Abstract

**Background:**

Many older patients approaching end-stage kidney disease have to decide whether to go for dialysis or non-dialytic conservative care (CC). Shared decision-making is recommended to align the treatment plan with the patient’s preferences and values. Little is known about older patients’ experiences with shared decision-making on dialysis or CC.

**Methods:**

We performed a survey study, in collaboration with the Dutch Kidney Patients Association, in 99 patients aged ≥70 years who had chosen dialysis (*n* = 75) or CC (*n* = 24) after a shared decision-making process involving an experienced multidisciplinary team.

**Results:**

Patients stated to be overall satisfied with the shared decision-making process (% with score 6–10 on 11-point Likert scale, dialysis versus CC: 93% vs. 91%, *P* = 0.06), and treatment decision (87% vs. 91%, *P* = 0.03). However, patients also reported negative experiences, especially those who had chosen dialysis. Such negative experiences were related to the timing, informing, and level of decision-making being shared. More patients who selected dialysis indicated to have felt forced to make a decision, mostly due to the circumstances, such as their deteriorating health or kidney function, or by their nephrologist (31% vs. 5%, *P* = 0.01). Also, patients who selected dialysis mentioned a perceived lack of choice as most common reason for choosing dialysis, and 55% considered their own opinion as most important rather than their nephrologists’ or relatives’ opinion compared to 90% of the patients who had chosen CC (*P* = 0.02). A subset of patients who had chosen dialysis still doubted their treatment decision compared to no patient who had chosen CC (17% vs. 0%, *P* = 0.03).

**Conclusions:**

Older patients reported contrasting experiences with shared decision-making on dialysis or CC. Despite high overall satisfaction, the underlying negative experiences illustrate important but modifiable barriers to an optimal shared decision-making process.

**Electronic supplementary material:**

The online version of this article (10.1186/s12882-019-1423-x) contains supplementary material, which is available to authorized users.

## Background

An increasing number of older patients has advanced chronic kidney disease (CKD) [1, 2]. These patients, who often are frail, have comorbid conditions and decreased independence [3, 4], are being considered for dialysis, which has become the most common treatment pathway for end-stage kidney disease in older patients. Dialysis is an intensive treatment that patients, their family, and clinicians might consider as too burdensome, outweighing the benefits. Non-dialytic conservative care (CC) has been recognized as reasonable alternative [5]. The main goal of a multidisciplinary CC pathway is to preserve quality of life instead of longevity. There is growing evidence from observational studies that dialysis may not prolong life or improve quality of life compared to CC in older patients, particularly in the oldest old and those with severe comorbidity [6–10].

Recent guidelines recommend shared decision-making as model to decide on preferred treatment in patients with advanced CKD [5, 11–14]. The main goal of shared decision-making is to align the treatment plan with the patient’s preferences and values by having discussions between patient and professional to come to a joint decision. Although the new guidelines help clinicians to do so, there is ongoing debate how the shared decision-making process on dialysis or CC should take place. For example, what timing is best to initiate decision-making [15–17], what factors influence patient and professional decision-making [16, 18], and how to properly counsel and involve older patients [17, 19–21]. Furthermore, in shared decision-making both patients and clinicians need to understand what considerations are important for the other.

The professionals’ perspectives on treatment decision-making in older patients with advanced CKD have been studied relatively well. These studies found that nephrologists attach most value to the patient’s preferences, followed by comorbidity, cognitive function, and physical function [22, 23]. Other studies, however, showed that nephrologists predominantly base the decision whether to start dialysis on biomedical factors and a tendency to prolong life [18], that they struggle to explain disease trajectory and prognosis [21, 24–26], and that nephrologists differ in their interpretation and approach to patient engagement [20].

Little is known about the views of older patients with advanced CKD on shared decision-making for choosing dialysis or CC. Several studies have explored reasons of older patients for their treatment choice [27–31], but only three included both patient groups who either had chosen dialysis or CC [32–34]. Furthermore, older patients’ experiences with, and preferences for, shared decision-making on dialysis or CC are still largely unexplored, particularly of patients who chose CC [16, 18]. Better understanding of these aspects for both patient groups may help to improve shared decision-making processes and patient-centered care. Therefore, the aim of our survey was to assess and compare older patients’ experiences with, and preferences for, a shared decision-making process on dialysis or CC.

## Methods

In collaboration with the Dutch Kidney Patients Association, two patient representatives and a policy adviser were involved in designing the study and questionnaire. They performed an anonymous systematic evaluation of our research protocol using an assessment form developed by the Association.

### Participants

Patients with stage 4/5 CKD aged ≥70 years who had chosen dialysis or CC after a shared decision-making process were recruited from a previously identified cohort in a non-academic teaching hospital in The Netherlands [8]. Patients of this cohort alive in 2015 and 2016 were asked to participate during a routine hospital visit or by phone. Exclusion criteria were mental incapacitation or language problems of such severity that the informed consent procedure or the questionnaire could not be completed. Written informed consent was obtained from all included patients. The local research ethics committee waived the need for ethical approval.

### Shared decision-making process

An experienced multidisciplinary team of nephrologists, nephrology nurses, social workers, and dieticians was involved in the shared decision-making process on preferred treatment. As part of standard care, the nephrologist initiated the process when the patient’s kidney function dropped < 20 mL/min/1.73m^2^ by making the patient and family aware of the need for a decision. This was followed by in-depth discussions with the patient and family on preferred treatment, during which oral and written information were given about practicalities, benefits, and risks of the different treatment modalities including dialysis and CC. Each shared decision-making process was tailored to the individual patient recognizing the patient’s needs and preferences in making a decision. Alongside the regular outpatient visits, standard but not obligatory components offered to all patients included a one-hour counselling and education session about possible treatment by the nephrology nurse, and a visit to the patient’s home by the social worker. Patients were also invited to visit the dialysis unit. Finally, a decision on preferred treatment was made during a consultation with the nephrologist, which was defined in the study as ‘original treatment decision’ and based on the recording note in the medical record. This decision was regularly evaluated and patients always had the opportunity to change their original decision.

In patients choosing hemodialysis or peritoneal dialysis, dialysis treatment was prepared and initiated once needed. We defined the dialysis group as all patients who had chosen for the intention to start dialysis after the shared decision-making process (ie, choice for dialysis-group), comprising both pre-dialysis patients and those who started with dialysis. In patients choosing CC, active medical treatment and multidisciplinary care were continued.

### Questionnaire

The questionnaire was newly developed to assess experiences and preferences of older patients related to their shared decision-making process on dialysis or CC, and to explore reasons for their treatment choice. Input on the questionnaire—including content, appropriateness of wording, and clarity—was generated from the literature and from the multidisciplinary team involved in counselling (nephrologists, nurses, social workers), an ethicist, two patient representatives, and a policy advisor of the Dutch Kidney Patients Association to establish content validity. Main topics to be assessed were the patient’s level of preparedness for shared decision-making, the timing of the decision-making, its informing, the level of decision-making being shared, and the patient’s satisfaction with the shared decision-making process and treatment decision. The final version consisted of 27 questions, including: binary questions (yes/no); questions with a 11-point Likert scale, categorised into: positive (score 6–10), neutral (score 5), or negative answer (score 0–4); and open-ended questions (Additional file 1). Additional questions were included to determine marital status, religion, and education level [35].

### Data collection and analyses

The questionnaire was completed by patients at a self-chosen moment, or administered by a researcher (WV, JD) during a hospital visit. Data collected from electronic medical records included: age, sex, comorbidity, time since original treatment decision (defined as time between recording note of original treatment decision after the shared decision-making process in the medical record, and taking part in the survey), and number of consultations about preferred treatment between patient and healthcare team. Comorbidity was scored according to the Davies comorbidity score [36], which is based on the presence of seven comorbid conditions producing three risk groups: no comorbidity (score 0), intermediate comorbidity (score 1 or 2), and severe comorbidity (score ≥ 3). Descriptive statistics were used including the unpaired *t*, chi-squared, Fisher-Freeman-Halton, and Mann-Whitney U test. A *P* value < 0.05 was considered statistically significant. Statistical analyses were performed using IBM SPSS Statistics 24.0. Open text responses were independently categorised into themes by two researchers (WV, JD), followed by discussions within the research team—including a patient representative—to reach consensus.

## Results

Of 128 eligible patients, 99 (77%) consented and answered the questionnaire: 75 who had chosen dialysis, and 24 CC after the shared decision-making process. The median time between original treatment decision and the survey was 19.4 months for patients of the dialysis group (interquartile range: 9.3–49.7 months), and 11.6 months for patients of the CC group (interquartile range: 3.8–30.2 months). Prior to the survey, two patients had changed their original decision to start dialysis into CC, and one patient from CC into hemodialysis. They were analysed according to their most recent decision on preferred treatment. Of the 75 patients who selected dialysis, 34 (55%) patients initiated dialysis in the period between original decision and the survey (median of 22.9 months between dialysis start and survey). In patients of the CC group, the median eGFR at the time of the survey was 15.0 mL/min/1.73m^2^ (interquartile range: 12.5–20.5 mL/min/1.73m^2^).

Table 1 shows the patient characteristics. Patients of the CC group were older, and lived more frequently without a partner. No significant differences were observed in sex, Davies comorbidity score, education level, and religion. Patients of the dialysis group had on average twice as many consultations about preferred treatment with the healthcare team during the shared decision-making process.

**Table 1:** Patient characteristics

	Choice for dialysis (*n* = 75)	Choice for conservative care (*n* = 24)	*P* value
Mean age (years)	79.8 ± 4.3	84.2 ± 4.9	< 0.001
Female	21 (28%)	11 (46%)	0.10
Davies comorbidity score			0.88
No comorbidity	9 (12%)	2 (8%)	
Intermediate comorbidity	44 (59%)	15 (63%)	
Severe comorbidity	22 (29%)	7 (29%)	
Currently living with partner	48/70 (69%)^a^	10/23 (44%)^a^	0.03
Stated to be religious	55/74 (74%)^a^	17 (71%)	0.74
Education level^b^			0.15
Primary education	16/73 (22%)^a^	10 (42%)	
Secondary education	44/73 (60%)^a^	10 (42%)	
Tertiary education	13/73 (18%)^a^	4 (17%)	
Time since original treatment decision			0.04
< 6 months	7 (9%)	8 (33%)	
6–12 months	18 (24%)	4 (17%)	
12–24 months	21 (28%)	5 (21%)	
> 24 months	29 (39%)	7 (29%)	
Current treatment modality	41 (55%) pre-dialysis 24 (32%) hemodialysis 10 (13%) peritoneal dialysis	24 (100%) conservative care	
Median number of consultations about preferred treatment between patient and healthcare team	4 (interquartile range: 3–5; minimum: 2; maximum: 14)	2 (interquartile range: 1–6; minimum: 1; maximum: 11)	0.002
Interviewer-administration of questionnaire	26 (35%)	6 (25%)	0.38

Values are numbers (%) unless stated otherwise

^a^the total number of patients is lower due to missing answers for this variable

^b^education level is based on the International Standard Classification of Education [53]

### Patients’ satisfaction

**Fig. 1:**
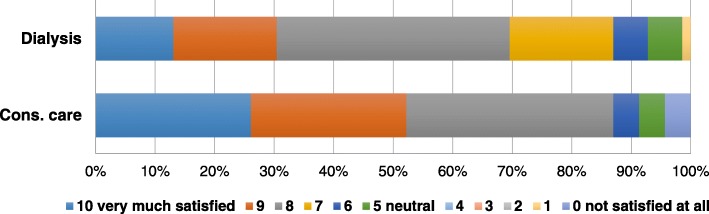
Older patients’ satisfaction with the shared decision-making process for choosing between dialysis and conservative care (*P* = 0.06). Rating on a 11-point Likert scale. Abbreviation: cons. care, conservative care

**Fig. 2:**
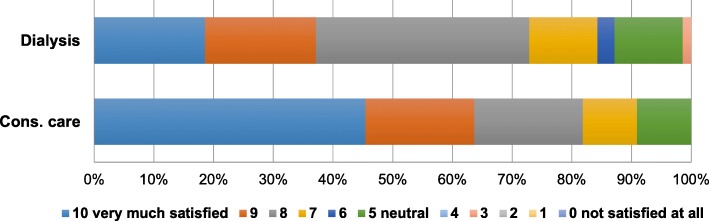
Older patients’ satisfaction with their treatment decision (*P* = 0.03). Rating on a 11-point Likert scale. Abbreviation: cons. care, conservative care

Figures 1 and 2 show the patients’ satisfaction with the shared decision-making process, and treatment decision rated on a 11-point Likert scale. The majority reported to be satisfied with their decision-making process (% with score 6–10, dialysis versus CC: 93% vs. 91%, *P* = 0.06), and treatment choice (87% vs. 91%, *P* = 0.03).

### Patients’ experiences with shared decision-making

Table 2 presents the findings on patients’ experiences with shared decision-making on dialysis or CC (for additional findings: see Additional file 2).

**Table 2:** Older patients’ experiences with the shared decision-making process on choosing dialysis or conservative care

	Choice for dialysis (*n* = 75)	Choice for conservative care (*n* = 24)	*P* value
Did you have prior thoughts on preferred treatment before counselling started?^a^	27/74 (37%)	7/24 (29%)	0.51
Do you think counselling was started at the right time?^a^	63/70 (90%)	19/21 (91%)	1.00
Do you think there was enough time to make a treatment decision?			0.66
Positive answer	57/72 (79%)	20/23 (87%)	
Neutral answer	14/72 (19%)	3/23 (13%)	
Negative answer	1/72 (1%)	0/23 (0%)	
Did you feel forced to make a decision?^a^	23/74 (31%)	1/22 (5%)	0.01
Was the possibility mentioned to postpone a decision?^a^	28/72 (39%)	15/21 (71%)	0.008
Do you think you had enough information to choose whether or not to start dialysis?			0.23
Positive answer	59/73 (81%)	16/22 (73%)	
Neutral answer	14/73 (19%)	5/22 (23%)	
Negative answer	0/73 (0%)	1/22 (5%)	
Did you miss information?^a^	4/63 (6%)	3/18 (17%)	0.18
Was withholding dialysis discussed as treatment option?^a^	36/72 (50%)	19/22 (86%)	0.002
Did you receive sufficient guidance?^a^	65/68 (96%)	20/21 (95%)	1.00
Did you feel supported by your doctor and/or nurse in your choice?			0.06
Positive answer	60/71 (85%)	16/22 (73%)	
Neutral answer	11/71 (15%)	4/22 (18%)	
Negative answer	0/71 (0%)	2/22 (9%)	
Did you feel supported by your partner and/or relatives in your choice?			0.74
Positive answer	56/66 (85%)	18/22 (82%)	
Neutral answer	10/66 (15%)	4/22 (17%)	
Negative answer	0/66 (0%)	0/22 (0%)	
How much confidence did you have in your doctor’s advice whether or not to start dialysis?			0.13
Positive answer	65/71 (92%)	18/23 (78%)	
Neutral answer	6/71 (9%)	5/23 (22%)	
Negative answer	0/71 (0%)	0/23 (0%)	
Whose opinion was most important in making your decision? (choose one)			0.02
Myself	34/62 (55%)	17/19 (90%)	
My nephrologist and/or nurse	22/62 (36%)	2/19 (11%)	
My partner and/or family	6/62 (10%)	0/19 (0%)	
Do you still have doubts about your treatment decision?^a^	12/70 (17%)	0/23 (0%)	0.03
Do you think decision-making could have been better?^a^	8/69 (12%)	4/19 (21%)	0.28

Values are numbers (%) of “yes” on a binary question^a^, or a positive (score 6–10), neutral (score 5), or negative answer (score 0–4) on a 11-point Likert scale. The total number of responses is indicated per question, excluding missing answers

^a^binary question (yes/no)

#### Preparedness for shared decision-making

The majority of both patient groups answered they had no prior thoughts on preferred treatment before counselling started (dialysis versus CC: 63% vs. 71%). They had never heard of dialysis before, perceived it as irrelevant to think about it yet: *“When there are no clinical signs or symptoms, it [treatment] is far from being on your mind” (dialysis patient)*, or had been postponing decision-making: *“[I had] previously thought about it, but [it] was not an issue yet, so [I] pushed it as far away as possible” (dialysis patient)*. Patients also described wrong expectations about the disease course: *“[I] had never thought it [dialysis] would become really necessary for me” (dialysis patient)*, or unawareness of their disease: *“[It] was a surprise to hear that my kidneys were functioning so badly” (CC patient).* Patients from the dialysis group who did have prior thoughts on preferred treatment experienced a lack of choice and did not see any alternatives than to undergo dialysis, while some were positive to do everything possible. Such patients from the CC group considered dialysis as too burdensome.

#### Timing of shared decision-making

Both patient groups reported that counselling on treatment plan was started at the right time (90% vs. 91%), and that there had been enough time to make a decision (79% vs. 87%). Those answering negatively would have preferred more time to consider their situation more extensively, to prepare themselves better: *“[Counselling] should have started much earlier, I was shocked when I heard about it [dialysis]. The doctor should have acted more in advance” (dialysis patient)*, or to still be able to actively try to delay further deterioration of their kidney function: *“I would have paid more attention to my health, with better diet. [I] would have been confronted with the consequences of my declining kidney function earlier” (dialysis patient)*, although some acknowledged that their clinical condition restricted time for decision-making. Four patients stated counselling had been started too early because dialysis initiation was still not needed, or they experienced stress about the potential start: *“[I have] mixed feelings, it [counselling] will always be unexpected. I know it for about a year now and think at each hospital visit: what are they going to say?” (choice for dialysis)*.

One third of the patients who had chosen dialysis reported they had felt forced to make a decision, compared to almost none of who had chosen CC (31% vs. 5%, *P* = 0.01). Most patients mentioned to have felt forced due to the circumstances, such as deteriorating health or kidney function, or by their nephrologist. Some mentioned their relatives, or a perceived lack of choice. Less patients of the dialysis group remembered if the possibility of postponing a decision was mentioned (39% vs. 71%, *P* = 0.008).

#### Informing shared decision-making

Both patient groups answered they had received enough information to make a decision (81% vs. 73%). However, relatively more patients who had chosen CC indicated to have missed information (6% vs. 17%, *P* = 0.18). They preferred more information about the different treatment options, the trajectory from preparation of dialysis treatment—including the shunt operation—until start, and possible symptoms at low kidney function.

Half of the patients who had chosen dialysis remembered that withholding dialysis was discussed as treatment option, against most patients who had chosen CC (50% vs. 86%, *P* = 0.002). One CC patient answered that treatment options other than CC were barely discussed.

#### Level of decision-making being shared

About half of the patients who had chosen dialysis indicated their own opinion as most important in making their treatment decision, compared to almost all patients who had chosen CC (55% vs. 90%, *P* = 0.02). Patients of the dialysis group more frequently reported the nephrologist’s or nurse’s opinion (36% vs. 11%), and of relatives (10% vs. 0%) as most important.

The majority reported they had experienced sufficient guidance from the healthcare team during decision-making (96% vs. 95%), and had confidence in their doctor’s advice on preferred treatment (92% vs. 78%). Those who experienced insufficient guidance mentioned they needed more time and information. One patient answered: *“In retrospect, I did not completely understand what dialysis is. An educational video using simple and comprehensible language would have been nice. Particularly because of [my] reduced capacity to process information due to my high age” (CC patient)*.

Most patients in both groups felt supported by their nephrologist and nurse in their decision (85% vs. 73%). Two patients of the CC group did not feel supported at all. Patients felt supported by their relatives (85% vs. 82%), although explaining their choice could be difficult: *“It took a lot of energy to explain our children that I deliberately opted for conservative care. At first, they did not understand it. Now they do” (CC patient).*

#### General suggestions for improvements

Subsets of both patient groups answered that decision-making could have been better (12% vs. 21%). Suggested improvements were: more information on all treatment options, tailoring of information to an individual’s situation, and more time, deliberation, and involvement in decision-making.

#### Current doubts about treatment choice

Twelve patients who had chosen dialysis answered to still have doubts about their treatment decision at the moment of the survey, compared to none of who had chosen CC (17% vs. 0%, *P* = 0.03). These patients were reconsidering their decision: *“Should I really do it [dialysis] considering my age?” (still pre-dialysis)*, or doubting which dialysis modality would be best: *“Whether or not to start dialysis is not difficult, but if I can do everything on my own is uncertain” (choice for peritoneal dialysis)*. One patient indicated to regret his decision to start dialysis: *“[I] do not know if I want to live longer, [I] would rather have died back then [before start]”*. Another patient did not doubt his decision for dialysis, although: *“If I would have to decide again: [I would choose to] withhold dialysis. [My] health has deteriorated a lot. Four years ago, I felt much more vital”.*

#### Reasons for treatment choice

Table 3 summarizes the patients’ responses to the open-ended question why they had chosen dialysis or CC. Both patient groups had contrasting reasons for their treatment choice: patients who had chosen dialysis most frequently mentioned a perceived lack of choice, and life prolongation; while patients who had chosen CC most frequently mentioned the treatment burden of dialysis, its impact on their quality of life, and their age and sense of life completion.

**Table 3:** Older patients’ reasons for their treatment choice. Categorisation of open text responses to the question: “Why did you choose to start or withhold dialysis?”

Choice for dialysis (*n* = 66)	Examples of answers
Lack of choice (*n* = 28)	*“Because I have to.”*
Dialysis perceived as unavoidable	*“What has to be done has to be done.”*
Seeing no alternatives	*“[I have] no other choice.”*
Rejecting conservative care as option	*“It [dialysis] was just necessary; withholding is no option.”*
Not eligible for other treatment options	*“I won’t receive a new kidney; I just have to [start dialysis], that’s just the way it is.”*
Life prolongation (*n* = 20)	*“[I] want to live longer.”*
Doing everything to prolong life	*“Withholding [dialysis] was no option, I still did not feel tired of life.”*
Enjoying life	*“[I am] far from finished being on this planet.”*
Social consideration	*“I just have to [start dialysis] for my partner and daughter.”*
To be longer with family	*“[I] want to live on, [I have] a lot of family.”*
To take care of ill partner	*“[My] husband has Alzheimer’s disease, [I] want to be there for him.”*
Following advice of doctor (*n* = 10)	*“On the nephrologist’s advice.”*
To maintain or improve quality of life or symptoms (*n* = 6)	*“[My] physical condition deteriorated, [I] wanted to remain active with table tennis.”*
Reconsidering or doubting choice (*n* = 6)	*“I sometimes think: should I really do it [dialysis]? Age 77 years. I consider to withhold dialysis.”*
Choice for conservative care (*n* = 21)	
High treatment burden of dialysis, particularly in-centre (*n* = 14)	*“[Dialysis means] too much hospital.”*
Too much impact on quality of life	*“No time to live normally any longer [with dialysis].”*
Loss of autonomy	*“I felt reluctant to live with dialysis. [I] still am an active woman, don’t want to be constrained.”*
Physical burden of dialysis	*“Potential side-effects of dialysis treatment.”*
Feeling well without dialysis	*“I still feel well, not ill.”*
Not eligible for or fearing burden of home dialysis	*“No option to go for home dialysis; home dialysis is probably disappointing.”*
High age and sense of life completion (*n* = 11)	*“[I] did not want it [dialysis], age of 84 years. [I] always thought: a human being should be allowed to just die!”*
Unlikely survival benefit of dialysis (*n* = 3)	*“No difference in life expectancy with or without dialysis.”*
Poor health (*n* = 2)	*“[My] health condition.”*
Stories of other patients (*n* = 2)	*“[A] visit to the dialysis unit and talking with patients were decisive factors [to choose conservative care].”*
Following advice of doctor (*n* = 2)	*“[My] nephrologist has given negative advice [to start dialysis].”*

## Discussion

In this survey, we determined older patients’ experiences with, and preferences for, shared decision-making on dialysis or CC. Patients indicated to be overall satisfied with their shared decision-making process, and treatment decision. However, we observed a discrepancy between the high satisfaction and underlying negative experiences that older patients reported as well, especially patients who had chosen dialysis. Such negative experiences were related to the timing, informing, and level of decision-making being shared. We found that a substantial subset of patients who had chosen dialysis still doubted their treatment decision. These findings show that—despite high overall satisfaction—older patients had contrasting experiences with shared decision-making on dialysis or CC, and the negative experiences illustrate important but modifiable barriers to an optimal shared decision-making process. We conclude that early initiation of decision-making is needed as in advance care planning and that shared decision-making should entail a dynamic process instead of a single point in time, including multiple interactions between patient, family and healthcare team about possible treatment and ongoing evaluation once a decision has been made.

Only few studies have assessed patients’ satisfaction with shared decision-making on dialysis or CC, or with the treatment decision. Seah et al. found all nine CC patients to be content with their decision, similar to our findings [30]. Ladin et al., however, observed low treatment satisfaction in dialysis patients who lacked engagement during decision-making [37]. Studies including ours also show that many patients who chose dialysis doubt or regret their decision, especially if the decision was more driven by the nephrologist’s preference [38–40]. No previous study directly determined patients’ satisfaction with the decision-making process, but several assessed patients’ experiences which give an indication. Consistent with our findings, patients often describe poor decision-making experiences, particularly patients on the most intensive treatment (dialysis) [16, 18, 32, 37]. Patients, including older patients, are found to desire more involvement in decision-making, which is associated with better outcomes like satisfaction, quality of life, and treatment adherence [19, 41–44].

The timing of shared decision-making is essential but there is ongoing debate what timing is best for decision-making processes on dialysis or CC [15–17]. Consistent with previous research [16, 19, 45], we found indications that decision-making should be initiated earlier because older patients felt unprepared or even forced to decide. More time and interactions with the healthcare team are needed to help patients understand and cope with their situation before a decision is to be made, to absorb information during decision-making, and to deliberatively weigh benefits and burdens of treatment options against their preferences and values. We think shared decision-making should therefore be approached as ongoing process which has to be initiated earlier rather than at a specific point in time when a decision on treatment becomes needed. Such early decision-making also gives the possibilities to postpone or reconsider a decision, which is preferred by older patients and could help those who doubt or regret their choice [29, 34, 39]. During our review of medical records for data collection, we found eight patients who had chosen dialysis at the time of the questionnaire to have changed their decision into CC; two had already received a dialysis shunt. Ongoing evaluation of a decision is important to assure if a chosen treatment pathway is still in line with the patient’s preferences as these may change, for example because of deteriorating health [19, 34, 46]. Care for older patients with advanced CKD offers valuable opportunities to timely start a dynamic process of shared decision-making because of the chronic disease course and often long-term relationships between patient and healthcare team.

To achieve decision-making to be shared, patients need to be aware that a decision is to be made and that their involvement matters. Studies including ours, however, frequently found that older patients are unaware of the need for a decision, experience a lack of choice—particularly patients who selected dialysis—and are not encouraged or enabled sufficiently enough to participate in shared decision-making [16, 18, 19, 37, 45]. We also found, consistent with previous studies, that only half of the dialysis patients indicated their own opinion as most important rather than their nephrologist’s [38–40], against the vast majority of CC patients [28, 30], while a similar approach to shared decision-making was applied in all patients. The findings in the dialysis group are in agreement with studies in different populations, such as patients with cancer or other chronic diseases, showing that patients frequently experience to lack a choice and that there is a mismatch between preference for and perceived participation in decision-making in about half of the patients [47–49]. The professional’s role is crucial in this but nephrologists are found to differ in their approach to shared decision-making and preferred level of patient involvement when choosing dialysis or CC [20, 21]. We think patient involvement should be individualized, recognizing differences in patients’ preferences for involvement [41, 42], though—as a minimum—clinicians should make every patient aware that a decision is to be made and that the patient’s opinion is important, to offer each patient the opportunity to become involved in the decision-making about their treatment.

Patients, and their family, need information about possible treatment options to be able to decide on treatment for end-stage kidney disease. Consistent with previous studies [32, 37–39, 50], however, we observed that many patients did not remember if withholding dialysis or CC was discussed, even reported by some patients on CC. This finding might indicate that CC was not discussed with patients, frequently found in other studies [21, 22, 32, 38, 50], or that it remains unclear to patients that CC could be chosen as treatment. Influencing factors are the nephrologist’s opinion about CC—some see CC as no care—and how well-established the CC pathway is in an institution [20, 21, 32]. Based on current evidence [5–10], CC is a reasonable treatment option in older patients. Therefore, patients should be informed about CC as one of the possible treatment options, including an explanation of the goals of a CC pathway to prevent misbeliefs that CC is the same as ‘doing nothing’.

Shared decision-making also involves the achievement to align a treatment decision with the patient’s preferences and values. Both patient groups are found to have contrasting reasons for their treatment choice: patients choose dialysis because of life prolongation, and CC because of quality of life [27–34]. As patients have different considerations, counselling should be tailored to each individual and incorporate the relevant topics for that patient. More data on patient-relevant outcomes of dialysis versus CC are needed [8, 51, 52]. Prognostic tools may be useful to inform patients, their family, and clinicians on possible outcomes, although tools need to be developed for both treatment pathways and should focus on not only survival but also other patient-relevant outcomes, like quality of life, symptoms and hospitalization [53]. More importantly, the findings on patients’ reasons for their treatment decision indicate that older patients with advanced CKD rather consider their values and goals towards life, quality of life, and death than having a biomedical focus including treatment effectiveness on which nephrologists base their decision [18, 20, 21]. A shift to a more person-centred ethos could facilitate better eliciting and understanding of patients’ priorities [19].

Our study has important limitations. First, recall bias may have influenced patients’ responses about their decision-making process; we cannot verify whether experiences did actually happen or how the process took place. Furthermore, the observed discrepancy between patients’ satisfaction but underlying negative experiences could be explained by socially desirable responding; true satisfaction might be lower. Second, the findings reflect the experiences and preferences of a limited number of patients. We think that each negative patient experience is relevant to take into account, although some were reported by a minority. Third, the generalizability of our results may be hindered by differences in approach to shared decision-making and CC at other institutions. Fourth, we used a self-developed questionnaire which needs further validation and focused on patients’ perspectives.

A strength is that we included both patient groups who either had chosen dialysis or CC. Except for age and living status, the groups were comparable; observed differences could not be explained by patient characteristics as comorbidity and education level. Another strength is our longstanding institutional policy to discuss both dialysis and CC with patients. Further exploration is needed to improve our understanding of patients’ experiences and preferences, as well as the role of patients’ partner and family in decision-making.

## Conclusions

We found that older patients with advanced CKD had contrasting experiences with shared decision-making on dialysis or CC alongside high overall satisfaction, and identified important barriers for improvement. We conclude that early initiation of decision-making is needed as in advance care planning and that shared decision-making should entail a dynamic process instead of a single point in time. Such approach to shared decision-making will help to achieve the overall goal to collaboratively decide by patient and professional on a treatment pathway that fits best with the patient.

## Additional files

Additional file 1: Questionnaire on patients’ experiences with, and preferences for, shared decision-making on dialysis or conservative care. (PDF 272 kb)

Additional file 2: Additional results of the questionnaire on older patients’ experiences with shared decision-making on dialysis or conservative care. (PDF 335 kb)

## Data Availability

The datasets used and/or analysed during the current study are available from the corresponding author on reasonable request.

## Abbreviations

CC: Conservative care
CKD: Chronic kidney disease
IBM SPSS: International Business Machines Statistical Package for the Social Science
Vs: versus

## References

[1] Couser WG, Remuzzi G, Mendis S, Tonelli M (2011). The contribution of chronic kidney disease to the global burden of major noncommunicable diseases. Kidney Int.

[2] Xie Y, Bowe B, Mokdad AH, Xian H, Yan Y, Li T (2018). Analysis of the global burden of disease study highlights the global, regional, and national trends of chronic kidney disease epidemiology from 1990 to 2016. Kidney Int.

[3] Kallenberg MH, Kleinveld HA, Dekker FW, van Munster BC, Rabelink TJ, van Buren M (2016). Functional and cognitive impairment, frailty, and adverse health outcomes in older patients reaching ESRD-A systematic review. Clin J Am Soc Nephrol.

[4] Berger JR, Hedayati SS (2012). Renal replacement therapy in the elderly population. Clin J Am Soc Nephrol.

[5] Davison SN, Levin A, Moss AH, Jha V, Brown EA, Brennan F (2015). Executive summary of the KDIGO controversies conference on supportive Care in Chronic Kidney Disease: developing a roadmap to improving quality care. Kidney Int.

[6] Brown MA, Collett GK, Josland EA, Foote C, Li Q, Brennan FP (2015). CKD in elderly patients managed without dialysis: survival, symptoms, and quality of life. Clin J Am Soc Nephrol.

[7] Verberne WR, Geers AB, Jellema WT, Vincent HH, van Delden JJ, Bos WJ (2016). Comparative survival among older adults with advanced kidney disease managed conservatively versus with Dialysis. Clin J Am Soc Nephrol.

[8] Verberne WR, Dijkers J, Kelder JC, Geers ABM, Jellema WT, Vincent HH (2018). Value-based evaluation of dialysis versus conservative care in older patients with advanced chronic kidney disease: a cohort study. BMC Nephrol.

[9] O'Connor NR, Kumar P (2012). Conservative management of end-stage renal disease without dialysis: a systematic review. J Palliat Med.

[10] Wongrakpanich S, Susantitaphong P, Isaranuwatchai S, Chenbhanich J, Eiam-Ong S, Jaber BL (2017). Dialysis therapy and conservative Management of Advanced Chronic Kidney Disease in the elderly: a systematic review. Nephron..

[11] Renal Physicians Association. Shared Decision-Making in the Appropriate Initiation of and Withdrawal from Dialysis (Clinical Practice Guideline). 2nd ed. Rockville; 2010.

[12] Williams AW, Dwyer AC, Eddy AA, Fink JC, Jaber BL, Linas SL (2012). Critical and honest conversations: the evidence behind the "choosing wisely" campaign recommendations by the American Society of Nephrology. Clin J Am Soc Nephrol.

[13] Farrington K, Covic A, Aucella F, Clyne N, de Vos L, Findlay A (2016). Clinical Practice Guideline on management of older patients with chronic kidney disease stage 3b or higher (eGFR <45 mL/min/1.73 m2). Nephrol Dial Transplant.

[14] Koncicki HM, Schell JO (2016). Communication skills and decision making for elderly patients with advanced kidney disease: a guide for nephrologists. Am J Kidney Dis.

[15] Rosansky SJ, Schell J, Shega J, Scherer J, Jacobs L, Couchoud C (2017). Treatment decisions for older adults with advanced chronic kidney disease. BMC Nephrol.

[16] Morton RL, Tong A, Howard K, Snelling P, Webster AC (2010). The views of patients and carers in treatment decision making for chronic kidney disease: systematic review and thematic synthesis of qualitative studies. BMJ..

[17] Mandel EI, Bernacki RE, Block SD (2017). Serious illness conversations in ESRD. Clin J Am Soc Nephrol.

[18] Hussain JA, Flemming K, Murtagh FE, Johnson MJ (2015). Patient and health care professional decision-making to commence and withdraw from renal dialysis: a systematic review of qualitative research. Clin J Am Soc Nephrol.

[19] Bunn F, Goodman C, Russell B, Wilson P, Manthorpe J, Rait G (2018). Supporting shared decision making for older people with multiple health and social care needs: a realist synthesis. BMC Geriatr.

[20] Ladin K, Pandya R, Perrone RD, Meyer KB, Kannam A, Loke R (2018). Characterizing approaches to Dialysis decision making with older adults: a qualitative study of nephrologists. Clin J Am Soc Nephrol.

[21] Ladin K, Pandya R, Kannam A, Loke R, Oskoui T, Perrone RD (2018). Discussing conservative management with older patients with CKD: an interview study of nephrologists. Am J Kidney Dis.

[22] van de Luijtgaarden MW, Noordzij M, van Biesen W, Couchoud C, Cancarini G, Bos WJ (2013). Conservative care in Europe--nephrologists' experience with the decision not to start renal replacement therapy. Nephrol Dial Transplant.

[23] van Loon IN, Boereboom FT, Bots ML, Verhaar MC, Hamaker ME (2015). A national survey on the decision-making process of dialysis initiation in elderly patients. Neth J Med.

[24] Schell JO, Patel UD, Steinhauser KE, Ammarell N, Tulsky JA (2012). Discussions of the kidney disease trajectory by elderly patients and nephrologists: a qualitative study. Am J Kidney Dis.

[25] Wachterman MW, Marcantonio ER, Davis RB, Cohen RA, Waikar SS, Phillips RS (2013). Relationship between the prognostic expectations of seriously ill patients undergoing hemodialysis and their nephrologists. JAMA Intern Med.

[26] van Biesen W, van de Luijtgaarden MW, Brown EA, Michel JP, van Munster BC, Jager KJ (2015). Nephrologists' perceptions regarding dialysis withdrawal and palliative care in Europe: lessons from a European renal best practice survey. Nephrol Dial Transplant.

[27] Ashby M, op't Hoog C, Kellehear A, Kerr PG, Brooks D, Nicholls K (2005). Renal dialysis abatement: lessons from a social study. Palliat Med.

[28] Johnston S, Noble H (2012). Factors influencing patients with stage 5 chronic kidney disease to opt for conservative management: a practitioner research study. J Clin Nurs.

[29] Noble H, Meyer J, Bridges J, Kelly D, Johnson B (2009). Reasons renal patients give for deciding not to dialyze: a prospective qualitative interview study. Dial Transplant.

[30] Seah AS, Tan F, Srinivas S, Wu HY, Griva K (2013). Opting out of dialysis - exploring patients' decisions to forego dialysis in favour of conservative non-dialytic management for end-stage renal disease. Health Expect.

[31] Chanouzas D, Ng KP, Fallouh B, Baharani J (2012). What influences patient choice of treatment modality at the pre-dialysis stage?. Nephrol Dial Transplant.

[32] Tonkin-Crine S, Okamoto I, Leydon GM, Murtagh FE, Farrington K, Caskey F (2014). Understanding by older patients of Dialysis and conservative Management for Chronic Kidney Failure. Am J Kidney Dis.

[33] Visser A, Dijkstra GJ, Kuiper D, de Jong PE, Franssen CF, Gansevoort RT (2009). Accepting or declining dialysis: considerations taken into account by elderly patients with end-stage renal disease. J Nephrol.

[34] Lovell S, Walker RJ, Schollum JB, Marshall MR, McNoe BM, Derrett S (2017). To dialyse or delay: a qualitative study of older new Zealanders' perceptions and experiences of decision-making, with stage 5 chronic kidney disease. BMJ Open.

[35] International Standard Classification of Education (ISCED 2011) [Available from: http://uis.unesco.org/en/topic/international-standard-classification-education-isced. Accessed 28 Jan 2019.

[36] Davies SJ, Russell L, Bryan J, Phillips L, Russell GI (1995). Comorbidity, urea kinetics, and appetite in continuous ambulatory peritoneal dialysis patients: their interrelationship and prediction of survival. Am J Kidney Dis.

[37] Ladin K, Lin N, Hahn E, Zhang G, Koch-Weser S, Weiner DE (2017). Engagement in decision-making and patient satisfaction: a qualitative study of older patients' perceptions of dialysis initiation and modality decisions. Nephrol Dial Transplant.

[38] Gilman EA, Feely MA, Hildebrandt D, Edakkanambeth Varayil J, Chong EY, Williams AW (2017). Do patients receiving hemodialysis regret starting dialysis? A survey of affected patients. Clin Nephrol.

[39] Davison SN (2010). End-of-life care preferences and needs: perceptions of patients with chronic kidney disease. Clin J Am Soc Nephrol.

[40] Berkhout-Byrne N, Gaasbeek A, Mallat MJK, Rabelink TJ, Mooijaart SP, Dekker FW (2017). Regret about the decision to start dialysis: a cross-sectional Dutch national survey. Neth J Med.

[41] Wolff JL, Boyd CM (2015). A look at person- and family-centered care among older adults: results from a National Survey [corrected]. J Gen Intern Med.

[42] Chewning B, Bylund CL, Shah B, Arora NK, Gueguen JA, Makoul G (2012). Patient preferences for shared decisions: a systematic review. Patient Educ Couns.

[43] Mechta Nielsen T, Frøjk Juhl M, Feldt-Rasmussen B, Thomsen T (2018). Adherence to medication in patients with chronic kidney disease: a systematic review of qualitative research. Clin Kidney J.

[44] Shay LA, Lafata JE (2015). Where is the evidence? A systematic review of shared decision making and patient outcomes. Med Decis Mak.

[45] Joseph-Williams N, Elwyn G, Edwards A (2014). Knowledge is not power for patients: a systematic review and thematic synthesis of patient-reported barriers and facilitators to shared decision making. Patient Educ Couns.

[46] Bratzke LC, Muehrer RJ, Kehl KA, Lee KS, Ward EC, Kwekkeboom KL (2015). Self-management priority setting and decision-making in adults with multimorbidity: a narrative review of literature. Int J Nurs Stud.

[47] Tariman JD, Berry DL, Cochrane B, Doorenbos A, Schepp K (2010). Preferred and actual participation roles during health care decision making in persons with cancer: a systematic review. Ann Oncol.

[48] Brom L, Hopmans W, Pasman HR, Timmermans DR, Widdershoven GA, Onwuteaka-Philipsen BD (2014). Congruence between patients' preferred and perceived participation in medical decision-making: a review of the literature. BMC Med Inform Decis Mak.

[49] Hirpara DH, Cleghorn MC, Sockalingam S, Quereshy FA (2016). Understanding the complexities of shared decision-making in cancer: a qualitative study of the perspectives of patients undergoing colorectal surgery. Can J Surg.

[50] Song MK, Lin FC, Gilet CA, Arnold RM, Bridgman JC, Ward SE (2013). Patient perspectives on informed decision-making surrounding dialysis initiation. Nephrol Dial Transplant.

[51] Parvez S, Abdel-Kader K, Pankratz VS, Song MK, Unruh M (2016). Provider knowledge, attitudes, and practices surrounding conservative Management for Patients with advanced CKD. Clin J Am Soc Nephrol.

[52] Grubbs V, Tuot DS, Powe NR, O'Donoghue D, Chesla CA (2017). System-level barriers and facilitators for foregoing or withdrawing Dialysis: a qualitative study of nephrologists in the United States and England. Am J Kidney Dis.

[53] Couchoud C, Hemmelgarn B, Kotanko P, Germain MJ, Moranne O, Davison SN (2016). Supportive care: time to change our prognostic tools and their use in CKD. Clin J Am Soc Nephrol.

