# Supplementary material for: Older patients’ experiences with a shared decision-making process on choosing dialysis or conservative care for advanced chronic kidney disease: a survey study
Source: BMC Nephrol. 2019 Jul 16;20:264. doi: 10.1186/s12882-019-1423-x (PMC6635995; doi:10.1186/s12882-019-1423-x)
Supplement: Supplementary file 1 — Questionnaire on patients’ experiences with, and preferences for, shared decision-making on dialysis or conservative care. (PDF 272 kb) [file 12882_2019_1423_MOESM1_ESM.pdf]

ADDITIONAL FILE 1:

Questionnaire on patients' experiences with, and preferences for, shared decision-making on dialysis or conservative care

---

1. What is your marital status?[a]
2. What is your education level?[b]
3. What is your religion?[c]
4. Have you ever thought whether you would like to start dialysis, before dialysis treatment was discussed in the hospital? *(yes/no)*

   If yes, how did you feel about dialysis? *(open text answer)*

   If no, do you know why you did not have thought about it yet? *(open text answer)*
5. With which health professional did you have the first discussion about whether or not to start dialysis (choose one)? *(categories: nephrologist; nephrology nurse; resident; general practitioner; other, namely…)*
6. Do you think that counselling about dialysis was started at the right time? *(yes/no)*

   If no, why? *(open text answer)*
7. With whom did you discuss treatment? *(categories: partner; relatives; friends; general practitioner; nephrology nurse; social worker; dietician; other patients; other, namely…)*
8. How many discussions about treatment do you think you have had with your healthcare team? *(estimated number)*
9. How much time did it take between the first discussion about treatment and the final decision? *(estimated time; free to choose unit of time)*
10. What was your final treatment decision? *(categories: dialysis, conservative care)*
11. Why did you choose to start or withhold dialysis? *(open text answer)*
12. Which medical factors played a role in your treatment choice? *(categories: prognosis with or without dialysis; quality of life with or without dialysis; your age; presence of comorbidities; other, namely…)*
13. Do you think there was enough time to make an adequate decision? *(11-point Likert scale)*
14. Did you feel forced to make a decision? *(yes/no)*

    If yes, why? *(open text answer)*

15. Did you feel supported by:
    a. your nephrologist and/or nurse in your decision whether or not to start dialysis? *(11-point Likert scale)*
    b. your partner and/or relatives in your decision whether or not to start dialysis? *(11-point Likert scale)*
16. How much confidence did you have in your nephrologist's advice whether or not to start dialysis? *(11-point Likert scale)*
17. Whose opinion has been most important in making your decision (choose one)? *(categories: own opinion; partner; relative; friend; nephrologist; nephrology nurse; social worker; dietician; general practitioner; other, namely…)*
18. Who explained dialysis treatment to you? *(categories: nephrologist; nephrology nurse; social worker; dietician; general practitioner; patient association; other, namely…)*
19. In what way did you receive information about dialysis? *(categories: oral information; written information; video or film; websites; decision aid; other, namely…)*
20. Did you visit the dialysis unit during decision-making? *(yes/no)*
21. Have different treatment options been discussed during decision-making about dialysis? *(yes/no)*
22. Was withholding dialysis discussed as treatment option? *(yes/no)*

    If yes, who mentioned it first (choose one)? *(categories: nephrologist; yourself; nephrology nurse; social worker; general practitioner; other, namely…)*
23. Was the possibility to postpone a decision mentioned? *(yes/no)*
24. Do you think you have received enough information to choose whether or not to start dialysis? *(11-point Likert scale)*
25. Did you miss information? *(yes/no)*

    If yes, what information? *(open text answer)*
26. Are you satisfied with the decision-making process? *(11-point Likert scale)*
27. Do you think that decision-making could have been better? *(yes/no)*

    If yes, how? *(open text answer)*
28. Did you receive sufficient guidance? *(yes/no)*

    If no, what did you have missed? *(open text answer)*
29. Are you satisfied with your final decision to start or withhold dialysis? *(11-point Likert scale)*

If no, why? *(open text answer)*

30. Do you still have doubts about your decision? *(yes/no)*

If yes, about what? *(open text answer)*

---

[a]recategorised into: currently living with partner *(yes/no)*.

[b]recategorised into: stated to be religious *(yes/no)*.

[c]recategorised into: primary, secondary, or tertiary education—based on the International Standard Classification of Education [53].
