# Supplementary material for: Older patients’ experiences with a shared decision-making process on choosing dialysis or conservative care for advanced chronic kidney disease: a survey study
Source: BMC Nephrol. 2019 Jul 16;20:264. doi: 10.1186/s12882-019-1423-x (PMC6635995; doi:10.1186/s12882-019-1423-x)
Supplement: Supplementary file 2 — Additional results of the questionnaire on older patients’ experiences with shared decision-making on dialysis or conservative care. (PDF 335 kb) [file 12882_2019_1423_MOESM2_ESM.pdf]

ADDITIONAL FILE 2:

Table A1. Additional results of the questionnaire on older patients' experiences with shared decision-making on dialysis or conservative care

	**Choice for dialysis** (n=75)	**Choice for conservative care** (n=24)
With which health professional did you have the first discussion about whether or not to start dialysis (choose one)?		
Nephrologist	57/68 (84%)	20/23 (87%)
Nephrology nurse	9/68 (13%)	1/23 (4%)
General practitioner	0/68 (0%)	1/23 (4%)
Resident	0/68 (0%)	0/23 (0%)
Other	2/68 (3%)	1/23 (4%)
With whom did you discuss treatment?		
Partner	41/72 (57%)	8/24 (33%)
Family	41/72 (57%)	16/24 (67%)
Friends	8/72 (11%)	5/24 (21%)
General practitioner	8/72 (11%)	2/24 (8%)
Nephrology nurse	24/72 (33%)	7/24 (29%)
Social worker	9/72 (13%)	3/24 (13%)
Dietician	15/72 (21%)	3/24 (13%)
Other patients	2/72 (3%)	3/24 (13%)
No one else	4/72 (6%)	4/24 (17%)
How many discussions about treatment do you think you have had with your healthcare team? *(estimated number)*	median 2 (IQR 2–4, range 0–10, n=63)	median 2 (IQR 1–4, range 0–15, n=20)
How much time did it take between the first discussion about treatment and the final decision? *(estimated time, free to choose unit of time)*	median 1 month (IQR 0–6, range 0–120[a], n=47)	median 0 months [b] (IQR 0–2, range 0–3, n=17)
Which medical factors played a role in your treatment choice?		
Prognosis with or without dialysis	34/55 (62%)	8/24 (33%)
Quality of life with or without dialysis	28/55 (51%)	12/24 (50%)
Your age	23/55 (42%)	17/24 (71%)
Presence of comorbidities	8/55 (15%)	7/24 (29%)
Other	2/55 (4%)	2/24 (8%)

Who explained dialysis treatment to you?		
Nephrologist	61/75 (81%)	18/23 (78%)
Nephrology nurse	57/75 (76%)	7/23 (30%)
Social worker	11/75 (15%)	2/23 (9%)
Dietician	10/75 (13%)	2/23 (9%)
General practitioner	1/75 (1%)	0/23 (0%)
Patient association	1/75 (1%)	1/23 (4%)
Other	1/75 (1%)	3/23 (13%)
In what way did you receive information about dialysis?		
Oral information	71/74 (96%)	18/23 (78%)
Written information	37/74 (50%)	5/23 (22%)
Video or film	4/74 (5%)	6/23 (26%)
Internet	3/74 (4%)	0/23 (0%)
Decision aid	2/74 (3%)	0/23 (0%)
Did you visit the dialysis unit during decision-making?[c]	45/72 (63%)	7/23 (30%)
Have different treatment options been discussed during decision-making about dialysis?[c,d]	56/74 (76%)	9/22 (41%)
Who mentioned withholding dialysis first (choose one)?[e]		
Nephrologist	23/34 (68%)	10/16 (63%)
Myself	6/34 (18%)	5/16 (31%)
Nephrology nurse	4/34 (12%)	1/16 (6%)
Other	1/34 (3%)	0/16 (0%)

Values are numbers (%) unless stated otherwise. The total number of responses is indicated per question, excluding missing answers.

[a]one patient answered 10 years.

[b]eight patients answered they had needed no time.

[c]binary question (yes/no).

[d]the result on this question was not further analyzed due to different interpretations of the question (eg, ‘different treatment options’ was seen as different dialysis modalities only, or included conservative care as well).

[e]follow-up question on “Was withholding dialysis discussed as treatment option? *(yes/no)”.*

Abbreviation: IQR, interquartile range.
